# Safety and Feasibility of Blockade of NK Group-2 Member-A Receptor in Natural Killer Cells Combined with Cetuximab Antibody in Patients with Advanced Gastric Adenocarcinoma

**DOI:** 10.34172/apb.43859

**Published:** 2025-02-09

**Authors:** Maryam Samareh-Salavatipour, Shirin Tavakoli, Maryam Barkhordar, Iman Seyhoun, Nasim Vousooghi, Mohammad Vaezi, Afshin Ghaderi, Tahereh Bakhtiari, Ardeshir Ghavamzadeh, Javad Verdi, Mohammad Ahmadvand

**Affiliations:** ^1^Department of Applied Cell Sciences, School of Advanced Technologies in Medicine, Tehran University of Medical Sciences, Tehran, Iran; ^2^Cell Therapy and Hematopoietic Stem Cell Transplantation Research Center, Research Institute for Oncology, Hematology and Cell Therapy, Tehran University of Medical Sciences, Tehran, Iran; ^3^Department of Internal Medicine, Hematology and Medical Oncology Ward, Yasuj University of Medical Sciences, Yasuj, Iran; ^4^Department of Immunology, School of Medicine, Isfahan University of Medical Sciences, Isfahan, Iran

**Keywords:** Stomach neoplasms, Natural killer cells, Receptors, Monalizumab, Immune checkpoint proteins, Cetuximab

## Abstract

**Purpose:**

Blocking of inhibitory receptors such as NK group-2 member-A (NKG2A) enhances tumor immunity of natural killer (NK) cells. Additionally, antibody-dependent cellular cytotoxicity (ADCC) is an important cytotoxic modality of action of NK cells, which act as a functional bridge between innate and adaptive immunity. Here, we investigated the safety and feasibility of anti-NKG2A antibody-pretreated NK cells combined with IgG1 antibody (cetuximab) in patients with advanced gastric adenocarcinoma (GAC).

**Methods:**

In this pilot study, treatment was initiated with cetuximab-based chemotherapy, followed by three times adoptive administration of anti-NKG2A pretreated NK cells (at doses 7×10^8^ cells/injection) at 5-day intervals in three unresectable and locally advanced GAC patients who enrolled regarding vital signs and clinical characteristics. The clinical signs, laboratory parameters, and CTCAE (Common Terminology Criteria for Adverse Events) were documented for a safety and feasibility assessment.

**Results:**

The expanded cells were confirmed to be enriched in NK cells with high expression of CD56 (88.1%) and low expression of NKG2A (0.22%). The combination NK cell therapy was well tolerated, with transient adverse events. All patients were alive at the last follow-up (24 weeks). All patients showed overall decreases in tumor size and CA 19–9 level 4 weeks after combination therapy. However, two patients showed progressive disease (PD) after 12 weeks and the level of CA19-9 was increased in all three patients after 24 weeks.

**Conclusion:**

In conclusion, this study demonstrated the safety and feasibility of infusing high doses of anti-NKG2A pretreated NK cells combined with cetuximab in patients with GAC.

## Introduction

 Gastric cancer (GC) is the second most prevalent cancer globally.^1^ Ninety percent of stomach tumors are malignant, and gastric adenocarcinoma (GAC) constitutes 95% of all gastric malignancies.^2^ The overall 5-year survival rate is typically between 10% and 30% for patients with resectable GAC.^1,3^ Most cases of GAC are treated with surgical excision, total or subtotal gastrectomy, and lymphadenectomy.^4^ However, chemotherapy and radiotherapy are extremely effective.^5^ These approaches, however, are generally accompanied by the adverse effects of damaging normal tissues, programmed cell death, and interference with cellular functions.^6^ Very often, more advanced modalities like molecular targeted therapy, immunotherapy, and neoadjuvant chemotherapy have been proposed for GAC.^7^ In particular, immunotherapy has become a standard of care in malignant disease over the last several years, with significant clinical benefits in select populations. As such, NK cells are prime candidates for immunotherapy.^8^ They are distinguished from T cells because NK cells do not exhibit human leukocyte antigen (HLA) restriction and they swiftly recognize and eradicate their tumor targets. They have been shown to induce a graft-versus-tumor effect without causing graft-versus-host complications.^9^

 NK cell cytotoxicity relies on maintaining a balance between inhibitory and activating signals, which influence the release of granzyme and perforin, ultimately leading to tumor target apoptosis.^10^ Strategies to restore or replace dysfunctional NK cells offer an attractive therapeutic approach for GAC. Initial studies involving autologous NK cells failed to demonstrate significant clinical benefits, largely because of signals from inhibitory receptors on the surface of NK lymphocytes that frequently resulted in severe side effects.^11^ A well-established and key cytotoxic mode of action for NK cells is antibody-dependent cellular cytotoxicity (ADCC), which serves as a functional link between innate and adaptive immunity.^12^ Following this manner, blockade of checkpoint pathways, in particular, immune checkpoint blockade by targeting the inhibitory receptors and/or their ligands, such as NK group-2 member-A (NKG2A) and its ligand (HLA-E), may represent a potent strategy.^13-15^ Regarding GAC, a recent study exhibited that NKG2A ^+^ NK cells have exhausted phenotype and lead to a poor prognosis in patients.^16^ Inhibition of NKG2A-HLA-E pathway has the unique ability to enhance the effect of multiple receptors on NK cells, promoting long-term antitumor responses and enhancing the ability of NK cytotoxicity in the tumor microenvironment through non-redundant and/or complementary pathways.^17^ As a single or combination therapy, blockade of the NKG2A pathway will enhance the antitumor activity of NK cells in cancer patients. Against *in vitro/in vivo* GAC models, blockade of NKG2A demonstrated increased activation, decreased inhibitory signaling pathways, and increased expression of important transcription factors in NK cells.^18^ Humanized anti-NKG2A blocking monoclonal antibodies have demonstrated the ability to enhance degranulation and interferon-γ (IFN-γ) production by NKG2A ^+^ NK cells when encountering HLA-E ^+^ target cells.^19^ Additionally, NKG2A inhibition and its combination with antibody coating of tumor cells amplifies NK cell-mediated ADCC.^18,20^ Conversely, this information could suggest that an interesting investigation would be to study the combined effect of anti-NKG2A monoclonal antibodies with other treatments in order to augment their beneficial effects. Therefore, combining NK therapy with other modalities has further clinical benefits, such as higher response rates and longer survival.

 Cetuximab (its brand name is Erbitux) is a monoclonal IgG1 antibody designed to target the epidermal growth factor receptor (EGFR).^21,22^ The anticancer activity of cetuximab may also significantly contribute to antibody-dependent cytotoxicity.^23^ In preclinical and clinical tumor models, cetuximab enhances the antitumor activity of cytotoxic drugs and radiotherapy.^24,25^ Promising results have been observed with cetuximab for treating EGFR-expressing metastatic colorectal cancer, both for initial treatment phases and for patients whose disease is resistant to other therapies.^26,27^ The combination of cetuximab antibody with chemotherapy, including irinotecan/5-FU/leucovorin (FOLFIRI) and weekly oxaliplatin/5-FU/leucovorin (FUFOX), showed positive outcomes as a primary treatment option for advanced gastric or gastroesophageal junction adenocarcinoma in phase II trials.^28,29^ Patients with GAC exhibiting EGFR expression and low levels of ligands experienced better results when treated with cetuximab/mFOLFOX6.^30,31^

 Due to the aforementioned benefits, we investigated the safety and toxicity of the administration of anti-NKG2A-pretreated NK cells, along with cetuximab antibody, to improve ADCC function for the management of advanced GAC.

## Materials and Methods

###  Eligibility

 Following obtaining the consent form, three unresectable and locally advanced GAC patients were enrolled in this study between November 2, 2023 and February 22, 2024. The included patients were eligible based on the following criteria: histologically diagnosed with a diffuse form of GAC, failed prior standard therapy, having the same tumor size and prior treatment protocol, age > 20 and < 60 years, Eastern Cooperative Oncology Group (ECOG) performance status of 2 or lower, life expectancy of at least 3 months, not having serious cardiovascular disease, satisfactory functioning of vital organs, and normal blood test results as leukocyte count ≥ 3000/mm^3^ and ≤ 12000/mm3, neutrophil count ≥ 1500/mm^3^, platelet count ≥ 100,000/mm^3^, hemoglobin ≥ 9.0 g/dL, serum alanine aminotransferase (ALT) and aspartate aminotransferase (AST) ≤ 100 IU/L, serum total bilirubin ≤ 2 mg/dL, serum creatinine (Cr) ≤ 1.5 mg/dL, and blood urea nitrogen (BUN) level ≤ 25 mg/dL. The exclusion criteria were as follows: positive record for hepatitis B or C virus (HBV or HCV), human immunodeficiency virus (HIV), human T-lymphotropic virus-1 (HTLV-1), or syphilis infection, other active serious infection, major complications such as severe diabetes, unstable angina, or a heart attack within the last three months, pregnancy or lactation, a history of severe allergies or autoimmune disorders, or had received any type of cell therapy in the 6 months before inclusion.

###  Study design

 This was a non-blinded and non-randomized pilot study and received approval from the ethics committee at Tehran University of Medical Sciences (ethics code: IR.TUMS.TIPS.REC.1402.080). Furthermore, it was registered in the Iranian clinical trial data set (IRCT) as ID: IRCT20140818018842N36 on 09/12/2023. There was no preference in the selection of patients for dose administration, and patient enrollment was done in chronological order and was performed on a timeline from the beginning of the project. All participants were familiar with the study details. This multi-central study was designed and conducted by the Cell Therapy and Hematopoietic Stem Cell Transplantation Research Center of Shariati Hospital and the Department of Applied Cell Sciences of the School of Advanced Technologies in Medicine of Tehran University of Medical Sciences. NK cell preparation, patient enrollment, and clinical steps were performed at the Department of Oncology of Shariati Hospital.

###  Cell processing

 Three healthy women volunteered for this study at the ages of 29 and 38 with O^+^ blood types. The positive result for HIV, HBV, HCV, and blood culture was the exclusion criteria of donors. After signing informed consent, the isolation and expansion method was performed based on a previously described process.^32^ For this process, whole blood (30 mL from each donor) was obtained from random healthy unrelated donors with blood collection syringes and injectors, all contained within ethylenediaminetetraacetic acid (EDTA) Falcon tubes. Collected samples were promptly transferred to a clean room under cold conditions to separate peripheral blood mononuclear cells (PBMCs) using Ficoll Paque Premium (GE Healthcare’s, United States) gradient centrifugation. By negative selection, NK cell separation was performed with the help of a human NK cell isolation kit (Miltenyi Biotech, Germany). For NK cell expansion, 2 × 10^6^ CD3^+^ T-cell-depleted isolated cells were seeded in RPMI1640 (Gibco-USA) medium containing 5% HyClone fetal bovine serum (FBS, USA), 40 × 10^6^ 100 gamma-irradiated K562-modified feeder cells which expressing mbIl15, 4-1BBL (at a ratio of 1:20), and 1000 IU/mL of Magnetic Activated Cell Sorting (MACS) GMP-grade recombinant human interleukin-2 (IL-2, Miltenyi Biotec-USA). Every 3 days, a fresh medium supplemented with 1000 IU/mL of IL-2 and 10 ng/mL IL-15 was added to NK cells until they reached the appropriate cellular concentration after 21 days. The expanded cells were pooled and labeled in cell infusion bags containing 50 ml of normal saline (9%) and albumin (5%), and transported to the Department of Oncology of Shariati Hospital.

###  Quality control and characterization of isolated NK cells

 Bacterial and endotoxin contamination of the final and intermediate products was assessed using Gram staining and a kinetic turbidimetric Limulus amebocyte lysate (LAL) assay, respectively. Fungal and mycoplasma contamination was detected by culture using a MycoAlert Mycoplasma Detection Kit (Lonza Japan, Tokyo, Japan). The karyotype of the cells was checked after treatment with anti-NKG2A antibody. To assess the viability of the expanded cells, a trypan blue exclusion assay was utilized. The purity of peripheral blood-derived NK cells (PB-NK) was measured by quantitatively evaluating the proportions of CD3 - and CD56^+^ markers using flow cytometry (FACSCalibur Becton Dickinson, USA). Briefly, a total of 1 × 10^5^ cells were collected, washed with phosphate buffered saline (PBS), and stained with fluorochrome-labeled antibodies (CD56-APC and CD3-FITC, BioLegend, USA) for 20 min at room temperature and in the absence of light. After washing, the labeled cell percentages were determined using a flow cytometry instrument and FlowJo software for analysis.

###  In vitro cytotoxicity assay

 To assess the *in vitro* cytotoxic effect of human-activated NK cells (effector cells), the K562 cell line was chosen as the target cell line. Following cell counting, the effector (E) and target (T) cells were co-cultured in round-bottomed 96-well plates in ratios of 1:1 (E: 10^4^/100 μL, T: 10^4^/100 μL), 5:1 (E:5 × 10^4^/100 μL, T: 10^4^/100 μL), and 10:1 (E: 10^5^/100 μL, T: 10^4^/100 μL) in a final volume of 200 μL at 37°C and 5% CO_2_ for 4 h, followed by the measurement of the presence of lactate dehydrogenase (LDH) in the cell culture medium. The frequency of LDH is directly associated with the necrosis and cytotoxicity of NK cells. For cytokine analysis, the supernatants from the activated NK cells co-cultured with the K562 line for 24 hours were collected and analyzed for their content of IFN-γ and TNF-α using ELISpot kits from Sigma Aldrich (USA), following the guidelines provided by the manufacturer.

###  Treatment of NK Cells with anti-NKG2A antibody

 After evaluating NKG2A abundance on the NK cell surface, the cells were exposed to 4 mg/mL anti-NKG2A antibody. A decrease in the expression of the NKG2A receptor and cytotoxic activity of pretreated NK cells was investigated using flow cytometry and LDH assay, respectively.

###  Patient treatment protocol 

**Figure 1:**
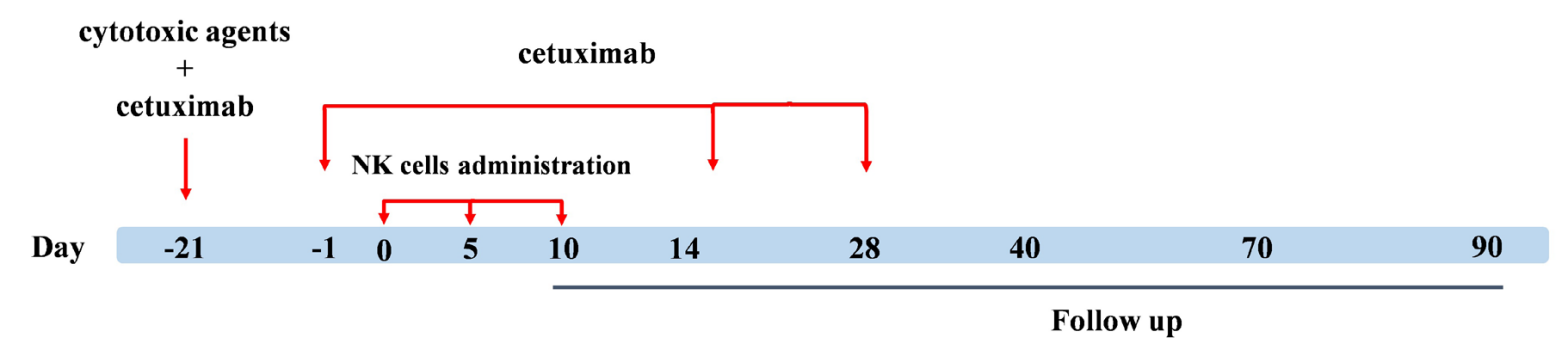
Treatment protocol. Expanded anti-NKG2A pretreated NK cells were intravenously injected every 5 days for three cycles. Additionally, three cycles of cetuximab antibody were administered biweekly. We looked at unfavorable occurrences over 28 days following the third cell injection. The tumor size and CA 19-9 expression level were checked before the initiation of therapy (baseline), 4 weeks (follow-up 1), 12 weeks (follow-up 2), and 24 weeks (follow-up 3) following the third dose of NK cells (follow-up 2)

 Cetuximab antibody was intravenously administered at a dosage of 400 mg/m^2^ on the day before the initial treatment cycle and was repeated at a dose of 250 mg/m^2^ every 2 weeks for a maximum of three doses. Fresh PB-NK cells (7 × 10^8^ cells) were intravenously infused into the patients over 60 min. The infusion was repeated every 5 days for 3 cycles, and the patients were monitored weekly during month 1 and biweekly during month 3 (Figure 1).

###  Assessment of clinical outcomes 

 The evaluation of safety and toxicity involved regular patient interviews, assessing of clinical conditions, vital signs, and laboratory tests. For this purpose, the National Cancer Institute Common Terminology Criteria for Adverse Events (CTCAE, version 5.0) checklist was used. It focuses on factors such as the risk of heart attack, variation in lung capacity, fever, rash, and anaphylactic shock. These symptoms were recorded using remote patient monitoring (RPM) devices from the beginning of infusion to discharge for 1 month. Furthermore, computed tomography (CT) scans were conducted using the Response Evaluation Criteria in Solid Tumors (RECIST VERSION 1.1) to evaluate the objective response of tumors both before and following the combination NK cell therapy.

 The count of blood cells containing white blood cells (WBC), red blood cells (RBC), and platelets was calculated before cell therapy (on the date of hospitalization) and 48 hours after the last cell infusion via an automated cell counter device (Sysmex, Japan). In addition, biochemical parameters, including alkaline phosphatase (ALP), ALT, BUN, Cr, and C-reactive protein (CRP), were investigated before the first cell injection and 48 hours after the last cell infusion using related kits. Importantly, the level of the CA 19-9 tumor marker was examined four times: before the beginning of cell therapy, 4, 12, and 24 weeks after the end.

###  Statistical analysis

 Data collected before and after the NK cell combination therapy were analyzed using the Wilcoxon signed-rank test. A P-value threshold of less than 0.05 was deemed statistically significant. The statistical evaluations were conducted utilizing GraphPad Prism 5 for Windows (GraphPad, San Diego, CA).

## Results and Discussion

 GAC ranks as the second most prevalent cancer globally.^1^ NK cells, as the primary component of innate immunity, can be a valuable adjunctive therapy; therefore, allogeneic off-the-shelf NK cell therapy holds promise in altering patients’ fate, safety, feasibility, and efficacy.^33,34^

 However, the restricted availability and infiltration, reduced cytotoxic efficacy of NK cells, and challenges related to tumor site suppression limit their therapeutic applicability for patients with GAC.^35,36^ Drawing on earlier studies that suggest inhibiting immune checkpoints may improve the effectiveness of NK cell treatments,^37,38^ this investigation examined the safety and effectiveness of an innovative combination therapy that includes cetuximab and NK cells that have been pre-treated with anti-NKG2A.

###  Patient characteristics

 For the first time, this study marks a pioneering effort to explore the impact of anti-NKG2A pretreated NK cells and cetuximab Ab on GAC patients. Combining activated NK cells and anti-NKG2A antibodies produced a synergistic therapeutic effect in xenograft models with HLA-E-expressing human cancer cells, according to a study by Ignacio Melero and colleagues.^39^ Furthermore, several preclinical and early-phase clinical trials have investigated the combination of NK cells and cetuximab in various malignancies, including colorectal and head and neck cancers.^40-42^ These studies have shown encouraging results, highlighting the potential of this combination to improve response rates and overall survival (OS).

 Between November 2023 and February 2024, three eligible patients with similar tumor sizes (two female (P-1 & P-3) and one male (P-2) were recruited for this study. The detailed patient characteristics are listed in Table 1. The mean age of the enrolled individuals was 52 years (range: 48-57). All patients had an ECOG performance status of 1 or less and showed local spreading (the median tumor size was 4.45 ± 0.7 cm). All three subjects had received the same chemotherapy regime before the start of the trial (including Oxaliplatin plus 5-FU/leucovorin) with no history of radiation therapy. A number of 7 × 10^8^ allogenic anti-NKG2A pretreated NK cells were administered thrice to a low population of patients. All of them completed their infusions without signs of disease progression during the administration period.

**Table 1:** Patient characteristics

**Case**	**Age/Gender**	**Diagnosis**	**ECOG**	**Disease Stage**	**Prior treatment**	**NK cell administration (3 infusions)**
P-1	48/F	Advanced gastric cancer	≤ 2	Locally advanced	2^nd^ line: Oxaliplatin plus 5-FU/leucovorin	Complete
P-2	52/M	Advanced gastric cancer	≤ 2	Locally advanced	2^nd^ line: Oxaliplatin plus 5-FU/leucovorin	Complete
P-3	57/F	Advanced gastric cancer	≤ 2	Locally advanced	2^nd^ line: Oxaliplatin plus 5-FU/leucovorin	Complete

Abbreviations: ECOG, Eastern Cooperative Oncology Group; Oxaliplatin, 5-fluorouracil and Leucovorin (FOLFOX-4).

###  Characterization of expanded NK cells

**Figure 2:**
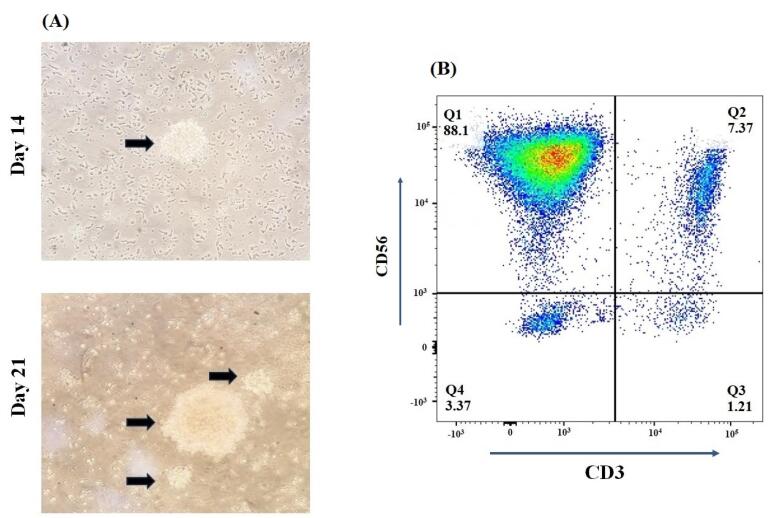
Expansion and Characterization dynamics of human isolated NK cells. (A) Arrows depict the shape of NK cells in round colonies after 14 and 21 days of cultivation.(B) The percentage of CD56 ^+^ CD3 - PBMC-derived NK cells was > 80% after MACS isolation

 In our study, isolated PBMCs revealed a median number of 15.38% (range 5.23–37.68) NK cells in total lymphocytes that reached an expansion rate of 621-fold (range 105-1225) after 21 days. From day 5 to day 21, clonal growth and round morphology were consistently observed (Figure 2A). The flow cytometry analysis of NK cell biomarkers (CD56^+^ and CD3^-^) demonstrated 88.1% (84.32-92.12) purity of expanded cells (Figure 2B). Furthermore, the evaluation of cell viability using trypan blue staining showed more than 95% viability before injection. In addition, the quality control tests revealed the absence of bacteria, fungi, mycoplasma, and endotoxins in the samples, which the reference laboratory further validated.

**Figure 3:**
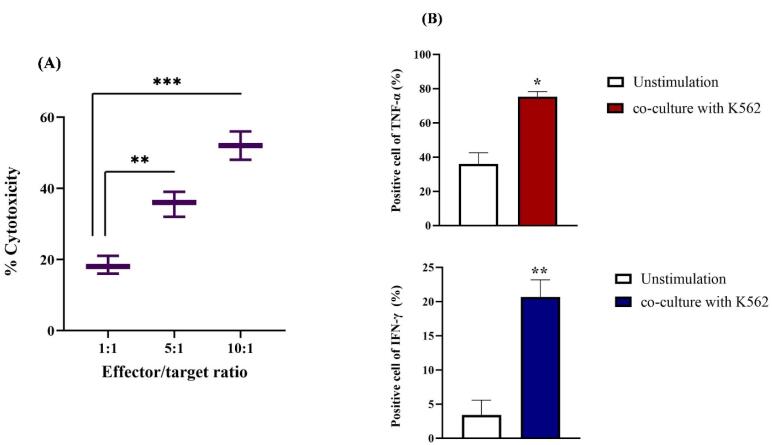
Cytotoxic activity and cytokine release of NK cells against K562 cell line. (A) Co-culturing of activated NK cells (E) and K562 cells (T) at different E: T ratios indicated the most cytotoxic effect in 10:1 E: T. (B) On the 21st day, the release of cytokines was measured in the stimulated NK cells, revealing that approximately 20% of interferon-gamma (IFN-γ) and 76% of tumor necrosis factor-alpha (TNF-α) were released. These levels were significantly higher compared to those in unstimulated NK cells. All results are expressed as means ± standard deviations (M ± SD, n = 3). Statistical significance was noted with **P ≤ 0.01 and ***P ≤ 0.001

 The cytotoxic activity of the final product was assessed using the K-562 cell line, an NK-sensitive target, and revealed significant cytotoxicity against these cells (Figure 3A). The strongest cytotoxicity was related to the 10:1 E: T ratio with a significant statistical difference (*P* < 0.001) from other ratios. Regarding cytokine release, we performed an ELISpot assay to quantify the secretion levels of two cytokines (TNF-α and IFN-γ) in NK cells co-cultured with the K-562 cell line. The results indicated a substantial rise in the release of IFN-γ (approximately 20%, *P*< 0.01) and TNF-α (around 76%, *P* < 0.001) compared to NK cells that had not been stimulated (Figure 3B).

###  Blocking of NKG2A 

 It has been reported that approximately 20-50% of circulating NK cells express NKG2A and approximately half of the expanded NK cells continued to express NKG2A under cGMP conditions.^43^ So, partial disruption of the axis of NKG2A and HLA-E can no longer suppress NK cells’ anti-tumor activity.

**Figure 4:**
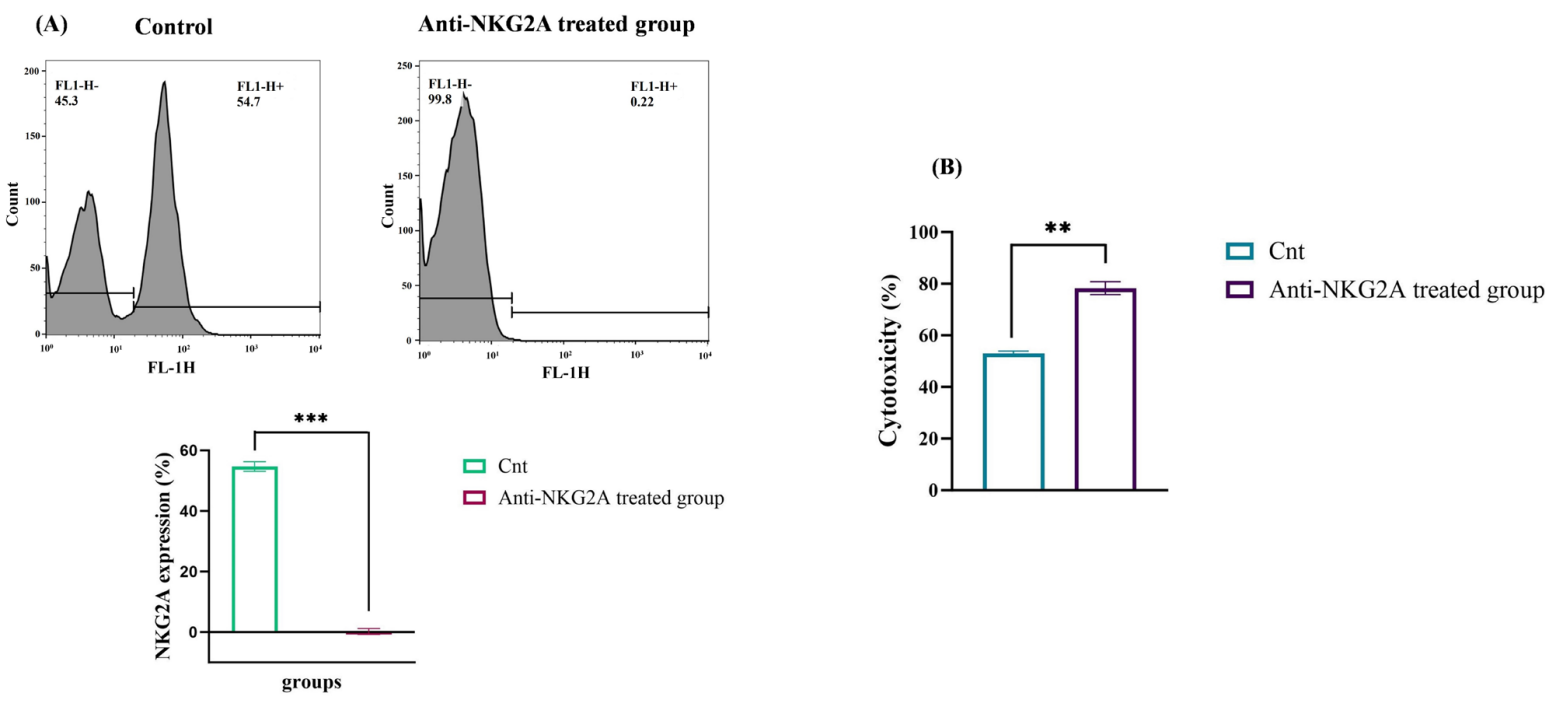
The effect of anti-NKG2A antibody on the expression of NKG2A receptor and cytotoxicity of NK cells. (A) Quantifying the level of NKG2A receptor demonstrated that treatment of activated NK cells with anti-NKG2A antibody significantly decreased the expression of NKG2A. (B) Following co-culturing with the K562 cell line at an E: T ratio of 10:1, anti-NKG2A-pretreated NK cells showed significantly higher cytotoxicity than the untreated NK cells. The findings are presented as mean ± standard deviation for three separate experiments. **P ≤ 0.01, and ***P ≤ 0.001

 Similarly, we indicated that treatment with anti-NKG2A decreased NKG2A expression and boosted NK cell cytotoxicity at various E/T ratios. The measurement of mean fluorescence intensity (MFI) revealed that treating NK cells with anti-NKG2A led to a remarkable decrease in NKG2A expression from 54.7% to 0.22 % (*P* < 0.001) (Figure 4A). Furthermore, to evaluate the cytotoxic effect of anti-NKG2A-pretreated human-activated NK cells in vitro, K562 cells were cocultured with NK cells. After a 24-hour incubation period, the cytotoxic effects of the NK cells on K562 were evaluated using the LDH assay. In the non-treated group, NK cells exhibited 53% cytotoxicity, while the group treated with anti-NKG2A demonstrated a significantly higher lysis rate of 78.3%. The remarkable antitumor activity of the activated NK cells was evident when NKG2A was blocked using the anti-NKG2A antibody (*P* < 0.01) (Figure 4B).

 NKG2A, a recently recognized immune checkpoint inhibitor expressed by cytotoxic lymphocytes, most prominently in NK cells.^44^ The combined action of the NKG2A receptor-HLA-E complex activates the intracellular phosphatases SHP-1 and SHP-2, inhibits the NKG2D activation signal, and promotes NK cell exhaustion.^44^ Therefore, anti-NKG2A antibodies are candidates for promising checkpoint inhibitors that target NK cells to enhance antitumor immunity.^45^ Alone, NKG2A blockade has shown limited therapeutic effectiveness, but it exhibits greater synergy with other immunotherapy-based agents. The most recent advances in tumor immunotherapy support a “combination” strategy, with NKG2A becoming recognized as the possible key regulator of innate and adaptive immune responses.^45-47^

 The overexpression of the EGFR in GC indicates that targeting the EGFR pathway may be a significant therapeutic strategy, as cetuximab markedly diminished the viability and proliferation of EGFR-positive tumor cells and reduced tumor volume and angiogenesis in a GC xenograft model. Studies have demonstrated that cetuximab inhibits the EGFR signaling pathway, which in turn inhibits tumor cell motility and invasion, cell survival, and cell cycle progression. Cetuximab causes cell death and reduces the synthesis of vascular endothelial growth factor (VEGF) and matrix metalloproteinase.^48^

 Additionally, cetuximab can cause ADCC by activating NK cells. On NK cells, NKG2A and CD94 form a heterodimer, and in cancer, especially GC, its ligand, HLA-E, is elevated. NKG2A inhibition improves cetuximab-induced NK cell ADCC and supports innate anti-tumor immunity mediated by NK cells. According to recent research, cetuximab and anti-NKG2A (monalizumab) together produced an excellent safety profile, a high response rate, a good duration of response, and encouraging progression-free survival, and OS.^49,50^

 Because the combination of cetuximab and monalizumab would induce ADCC, block EGFR, and disinhibit NK cells, they were used in several clinical studies.^50^ Cetuximab is a suitable choice for this combination therapy since it may be used to directly target tumor cells and enhance the activity of NK cells. It also has synergistic effects with monalizumab.

###  Safety assessment

 The primary focus of this research was to evaluate safety. Combining biological therapies can lead to unique side effects, such as infusion reactions or immune-related adverse events, necessitating careful monitoring and management. Although the safety of expanded allogeneic NK cells has been demonstrated in various clinical trials for GAC,^51-53^ this novel combination therapy has not been consistently evaluated for GAC.

 In this study, to facilitate the differentiation of adverse effects arising from chemotherapeutic agents and cetuximab antibody from those of expanded anti-NKG2A pretreated NK cell administration, subjects underwent a single regimen of chemotherapy with IgG1 antibody, followed by three cycles of NK cell combination therapy (Figure 1). CTCAE parameters like fever, rash, heart attack, respiratory changes, and anaphylactic shock symptoms were closely monitored over a 28-day period. Based on the CTCAE checklist, no severe adverse effects were observed in the patients. We detected transient symptoms in two patients, including mild tachycardia (3 occurrences of arrhythmia in one hour) and grade 1 fever, which were both negligible. The combination therapy did not increase the frequency or severity of toxicities. Therefore, the overall toxicity is shown in Table 2.

**Table 2:** Safety evaluation of the patients

**Patient**	**Fever**	**Rush**	**Heart attack**	**Bronchospasm**	**Edema**	**Allergic signs**	**Other adverse events**	**Arrhythmia**	**Safety assessment grading**
P-1	0	0	0	0	0	0	0	1	0
P-2	1	0	0	0	0	0	0	0	0
P-3	0	0	0	0	0	0	0	0	0

 Previous studies have shown that the (cetuximab and trastuzumab) is a safe and tolerable therapeutic strategy for the treatment of GAC and colon cancer.^52,53^ We demonstrated that the combination of anti-NKG2A pretreatment of NK cells with cetuximab antibody did not lead to an increase in toxic effect. The most frequently observed adverse reactions were mild fever and tachycardia. In conclusion, the anti-NKG2A pretreated NK cell therapy showed good tolerance and promising clinical responses and did not heighten the toxicities associated with cetuximab.

###  Clinical efficacy

 Although the evaluation of clinical outcomes was not the main focus of our experiment, there is limited information on the clinical response of patients.

 CT scans were conducted pre- and post-NK cell therapy to evaluate the response rate of patients. Data analysis revealed a not significant reduction in target lesion diameters in all patients at four weeks after NK cell administration compared to baseline. The clinical outcomes are summarized in Table 3, revealing the progressive condition of P-2 and P-3 due increase in the size of lesions during 12 and 24 weeks of follow-up. Although all patients showed stable disease (SD) after 4 weeks of follow-up, two showed signs of progressive disease (PD) 12 weeks after the third NK cell infusion. After 24 weeks, P-1 showed an increase in the level of CA19-9 which could be a marker of changing the disease status from SD to PD. Although the level of CA19-9 was increased in P-2 and P-3 after 24 weeks, it was not significant.

**Table 3:** Maximum clinical response per patient

**Patients**	**Response**				**Response rate**
	**CR**	**PR**	**SD **	**PD **	
**4 weeks follow-up**					
n = 3	-	-	3	0	three patients with stable disease
**12 weeks follow-up**					
n = 3	-	-	1	2	Two patients with PD, one patient with stable disease
**24 weeks follow-up**					
n = 3	-	-	-	3	three patients with PD

**Abbreviations: **CR, complete response; PR, Partial response; SD, stable disease; PD, progressive disease.

**Figure 5:**
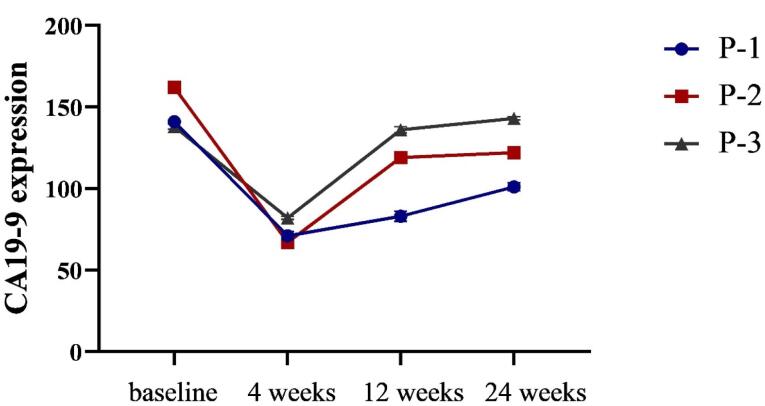
Serum levels of CA 19-9 before and following combination NK cell therapy. Administration of anti-NKG2A pretreated NK cells led to a decrease in CA 19-9 serum levels after 4 weeks in all 3 patients (not significant, *P* > 0.05). P-2 and P-3 showed an increase in the expression level of CA19-9 after 12 weeks (*P* > 0.05). Additionally, the level of CA19-9 was increased in P-1 after 24 weeks of follow-up (*P* > 0.05)

 As CA 19-9 has recently been shown to be a marker for cancers of the digestive tract, it was chosen for monitoring GAC patients in this study with a sensitivity of 47.7% and a specificity of 100% for GAC. CA19-9 is expected to be more beneficial than CEA for the detection of recurrent GC.^54,55^ To our surprise, infusion of NK cells led to a decrease in CA 19-9 serum levels after 4 weeks in all patients but increased after 12 weeks in 2 patients (P-2 and P-3, Figure 5). An increase in the level of CA19-9 after 12 and 24 weeks may be related to the genetic variation of patients that affects all aspect of NK cells. Furthermore, cancer cells’ resistance to apoptosis and the intricate relationship between the immune system and the tumor and its microenvironment are other mechanisms of resistance to NK cell therapy.^56^

 Several studies showed significant obstacles related to NK cell therapy in GAC including tumor infiltration, the suppression of tumor sites, and the alteration in the expression of activating and inhibitory receptors of NK cells.^37,57^ Thus, strategies to enhance the effectiveness of NK cells are important, and the use of immune checkpoint inhibitors may play a significant role in the enhancement of NK cell cytotoxicity.^37^ Narni-Mancinelli et al. indicated that NK cell education may enhance therapeutic efficacy,^58^ thereby opening new pathways for optimizing combination treatments which was consistent with our observations of increased cytotoxicity post NKG2A inhibition.

###  Laboratory parameters

 To assess organ functions, biochemical and hematological parameters were analyzed in blood samples from patients. Due to the administration high number of NK cells, the WBC count increased after 48 hours. The platelet and RBC count remained in normal range throughout the 48 hours after the last cell injection. The average international normalized ratio (INR) was 1.25 ± 0.29 on the first day of hospitalization, showing a non-significant increase 48 hours after the last NK cell infusion. So, this combination therapeutic strategy did not show a significant effect on the count of blood cells. The mean count of RBCs was approximately 5 million, showing no significant difference. Additionally, the daily measurement of serum concentration of BUN and Cr indicated not significant changes. Serum levels of liver factors, including ALP and ALT, remained stable throughout the 48-hour monitoring period. Also, the serum level of CRP did not change during the follow-up period. Laboratory hematological and biochemical information is described in Table 4.

**Table 4:** Descriptive data of laboratory parameters before and after combination NK cell therapy

**Parameters**	**Patients**					
	**P-1**		**P-2**		**P-3**	
	**Before**	**After**	**Before**	**After**	**Before**	**After**
WBC	6.2	7.55	5.8	7.3	7.6	9.1
RBC	4.3	4.5	6.1	5.9	4.8	5.1
Platelet count	140	158	180	164	210	195
BUN	12.4	14.6	14.3	14.9	18.2	17.6
Cr	0.95	1.02	1.15	1.09	0.97	1.02
ALP	235	245	224	219	287	295
ALT	32.4	31.5	18.7	18.2	35.7	36.3
CRP	8.3	8.32	9.4	9.1	8.8	8.2

 The levels of laboratory parameters were within the expected range for all patients. The preservation of normal liver function implies that the combination of cetuximab and pretreated NK cells did not disturb normal homeostasis. These findings are consistent with those of prior research on individuals undergoing adoptive NK cell therapy.^52,53^

 Allogenic NK cell therapy can prevent the onset of graft-versus-host disease through their cytotoxic capabilities, either by directly eliminating activated alloreactive T cells or indirectly by removing antigen-presenting cells (APCs).^37,59^ However, the simultaneous administration of anti-NKG2A antibody and NK cells is suggested to unleash patients’ NK and T cells to improve cytotoxic potential against intended cancer. Activated NK cells not only directly eliminate tumor cells but also enhance T cell proliferation by removing immune-suppressive cells and secreting cytokines.^60,61^ So, the synergistic application of mAbs like cetuximab demonstrates considerable promise. Additionally, studies have shown that NK cells can increase the cytotoxic effects of cetuximab *in vitro*, even when targeting RAS and BRAF mutant colorectal cancer cells.^40^ Therefore, it would be worth investigating the effects of combining anti-NKG2A pretreated NK cells and cetuximab in patients with GAC. Conversely, cetuximab can enhance the susceptibility of cancer cells to NK cell-induced killing by downregulating the expression of protective surface molecules, such as MHC class I, and upregulating death receptors.^62,63^ Moreover, cetuximab may facilitate the recruitment and activation of NK cells to the tumor site by modulating the tumor microenvironment.^64^

 Despite the promise of this combinatorial approach, challenges remain, such as optimization of NK cell isolation, expansion, and persistence in the patient’s body. Additionally, patient selection, based on biomarkers like EGFR expression and immune checkpoint status, will be crucial for maximizing treatment benefits. Further research is also needed to explore potential synergies with other immunotherapeutic agents and to understand the underlying immune mechanisms. In addition, future experiments must focus on expanding the samples and conducting phase I trials to rigorously evaluate the therapeutic effectiveness of this innovative approach in a larger cohort of patients with GAC.

## Conclusion

 For the first time, our pioneering study highlights the safety and feasibility of anti-NKG2A pretreated NK cells combined with cetuximab antibodies for treating GAC. We demonstrated the safety and feasibility of this novel therapeutic strategy in patients with advanced GAC. However, further studies should be considered for precise determination of the efficacy of this combination approach due to the absence of randomization, the low number of participants, and the short time of follow-up which limits the appropriate evaluation.

## Competing Interests

 The authors declare that they have no conflict of interest.

## Ethical Approval

 All procedures followed were in accordance with the ethical standards of the responsible committee on human experimentation (institutional and national) and with the Helsinki Declaration of 1964 and later versions. Informed consent to be included in the study, or the equivalent, was obtained from all patients.
